# Sestrin2 ameliorates diabetic retinopathy by regulating autophagy and ferroptosis

**DOI:** 10.1007/s10735-023-10180-3

**Published:** 2024-01-02

**Authors:** Xiaoting Xi, Qianbo Chen, Jia Ma, Xuewei Wang, Junyan Zhang, Yan Li

**Affiliations:** 1 Ophthalmology Department, The First Affiliated Hospital of Kunming Medical University, Kunming, Yunnan 650032 China; 2 Department of Clinical Epidemiology and Evidence-based Medicine, Shanxi Bethune Hospital, Shanxi Academy of Medical Sciences, Taiyuan, Shanxi 030000 China

**Keywords:** Sestrin2, Autophagy, Ferroptosis, Diabetic retinopathy

## Abstract

Diabetic retinopathy (DR) is a serious microvascular complication of diabetes. The aim of this study was to explore the effect of Sestrin2 on DR through the regulation of autophagy and ferroptosis levels and its mechanism. In vitro and in vivo DR models were established by high glucose (HG) and streptozotocin (STZ) induction of ARPE-19 human retinal pigment epithelial cells and C57BL/6 mice, respectively. In this study, we demonstrated that after HG treatment, the activity of ARPE-19 cells was decreased, the apoptosis rate was increased, endoplasmic reticulum (ER) stress was activated, autophagy levels were decreased, and ferroptosis levels were increased. Overexpression of Sestrin2 enhanced cell viability, reduced apoptosis and ferroptosis, and enhanced autophagy. However, the effect of overexpression of Sestrin2 was attenuated after the addition of the STAT3 phosphorylation activator Colivelin TFA (C-TFA), the mTOR pathway activator MHY1485 or the autophagy inhibitor 3-methyladenine (3-MA). In addition, the effect of Sestrin2 knockdown on cells was opposite to the effect of overexpression of Sestrin2, while the effect of Sestrin2 knockdown was attenuated after treatment with the ER stress inhibitor 4-phenylbutyric acid (4-PBA). Animal experiments also confirmed the results of cell experiments and attenuated the effects of overexpression of Sestrin2 after injection of the ferroptosis activators erastin or 3-MA. Our study revealed that Sestrin2 inhibits ferroptosis by inhibiting STAT3 phosphorylation and ER stress and promoting autophagy levels, thereby alleviating DR.

**Supplementary Information:**

The online version contains supplementary material available at 10.1007/s10735-023-10180-3.

## Introduction

Diabetic retinopathy (DR) is the most common complication of diabetes mellitus (DM) and one of the leading causes of blindness in the working population worldwide(Wei et al. 2022). DR is characterized by hyperglycemia, which causes changes in retinal microvascular function and integrity, leading to progressive retinal ischemia and angiogenesis (Deng et al. 2022). In the treatment of DR, the first is to control blood sugar or to delay the occurrence of DR by changing lifestyle, improving insulin resistance and repairing damaged islet cell function, or to treat DR by laser surgery, vitrectomy and other treatments (Deng et al. 2022; Liu et al. 2022a, b, c). However, the current treatment results of DR are often unsatisfactory, so it is necessary to explore more specific and precise molecular mechanisms to determine more effective prevention and treatment of DR.

Autophagy is a process that ensures the physiological turnover of aging and damaged cells under stress conditions, controls cell fate through various crosstalk signals, and maintains cellular homeostasis (Cao et al. 2014). Studies have shown that endoplasmic reticulum stress, oxidative stress, apoptosis and autophagy can induce retinal inflammation, leading to retinal angiogenesis and neuronal damage. Autophagy, as a major catabolic pathway for the degradation and recycling of damaged proteins or organelles, may be involved in the pathogenesis of DR (Wang et al. 2022). Autophagy in human retinal pigment epithelial cells (RPE) delays the occurrence of DR by regulating glycolipid metabolism and reducing oxidative stress, thereby reducing inflammation and clearing damaged mitochondria (Tanaka et al. 2012; Wang et al. 2017). Iron is important for the maintenance of homeostasis, and many physiological activities of the body can be regulated by regulating iron homeostasis (Jiang et al. 2021; Kerins and Ooi 2018). Ferroptosis is a novel iron-dependent programmed cell death characterized by fatal intracellular accumulation of iron and iron-induced lipid reactive oxygen species (ROS). Several mediators have been implicated in ferroptosis, including ROS accumulation, GSH depletion, and inhibition of GPX4 activity. In addition, FTH1, HO-1, and xCT also mediate ferroptosis (Hu et al. 2023). An increasing number of studies have shown that ferroptosis is involved in the occurrence of blinding diseases and the death process of retinal pigment epithelial cells induced by ROS (Chen et al. 2021b; Lee et al. 2020). In addition, ferroptosis is the cell death pathway of retinal vascular endothelial cells in DR, and inhibiting ferroptosis has an alleviating effect on diabetic complications (Fan et al. 2022; Zhang et al. 2021). Studies have demonstrated that autophagy regulates ferroptosis by regulating cellular iron homeostasis and cell ROS production (Gao et al. 2016). For example, activation of autophagy mediates ferroptosis by degrading the cellular ferriogen protein FTH1 (Zhang et al. 2020). In the RPE of diabetic rats, abnormalities in the autophagy‒lysosome degradation process lead to ACSL4 protein accumulation, which catalyzes the production of lethal lipid species and ultimately induces ferroptosis in RPE cells (Liu et al. 2022a). These studies suggest that autophagy and ferroptosis can affect the occurrence and development of DR, and their underlying mechanisms still need to be further explored.

Sestrin2, a member of the Sestrin protein family, is a highly conserved stress-induced metabolic protein. Sestrin2 has a dual function, which can directly reduce oxidative stress by restoring peroxiredoxin peroxide and indirectly reduce oxidative stress by regulating mTOR to enhance autophagy activity (Chen et al. 2021a). Sestrin2 plays a key role in various cell signal transduction processes, and its dysregulation has been linked to various diseases (Che et al. 2021). For example, calycosin has an inhibitory effect on papillary thyroid cancer by promoting apoptosis and autophagy through the Sestrin2/AMPK/mTOR pathway (Qu et al. 2022), and studies have shown that Sestrin2 is involved in the regulation of ferroptosis. Sestrin2 alleviates the development of sepsis by inhibiting ferroptosis of dendritic cells in sepsis (Li et al. 2021). In addition, research has found that Tricin alleviates DR by regulating Sestrin2/Nrf2 signaling to inhibit oxidative stress and angiogenesis (Yang and Li 2023). In addition, Sestrin2 has important clinical functions in a variety of metabolic diseases, such as diabetes and its complications, which can reduce insulin resistance by regulating glucose and lipid homeostasis, and studies have shown that serum Sestrin2 levels are significantly reduced in obese children and diabetic nephropathy patients (Lee et al. 2012; Mohany and Al Rugaie 2020; Nourbakhsh et al. 2017). Based on the above studies, we conclude that Sestrin2 may regulate the pathogenesis of DR by regulating autophagy and ferroptosis. Therefore, this study will analyze the effect of Sestrin2 on DR and its mechanism at the cellular and animal levels.

## Materials and methods

### Cell culture

The human retinal pigment epithelial (RPE) cell line ARPE-19 was purchased from Otwo Biotechnology Co., Ltd. (Shenzhen, China). ARPE-19 cells were cultured in Dulbecco’s modified Eagle medium (DMEM-F12) (ATCC, Manassas, USA) supplemented with 2 mmol/L glutamine, 10% fetal bovine serum and 100 U/mL penicillin/streptomycin and incubated in an incubator at 37 °C and 5% CO_2_. The cell culture medium was changed every two days. When cells reached 80% confluence, the cells were digested with trypsin and then inoculated in porous plates for subsequent experiments. Before the experiment, the cells were washed once with phosphate buffer (PBS), then ARPE-19 cells were induced with a high glucose concentration (HG, 25 mM) for 48 h, and the control cells were cultured in a medium containing normal glucose (NC, 5.5 mM)(Fan et al. 2022).

### Cell transfection

The expression vectors, oe-Sestrin2 and si-Sestrin2 (see supplementary file 1 for the sequences), were designed and synthesized by GeneChem (Shanghai, China). The coding sequences for the mRNAs of Sestrin2 were cloned into pcDNA3.1. Oe-Sestrin2 and si-Sestrin2 were transfected into cells with Lipofectamine®3000 reagent (Invitrogen, CA, USA), and transfection efficiency was measured.

### Cell viability test

Cell viability was measured using a CCK-8 assay kit as previously described (Liu et al. 2020). ARPE-19 cells were inoculated into 96-well plates at a density of 1 × 10^5^ cells/well and placed in a 5% CO_2_ incubator at 37 °C for 24 h. CCK-8 reagent (10 µL/well) was added, and the cells were cultured for 2 h. The 96-well plate was then placed on a microplate reader, and the absorbance of the cells at 450 nm was measured.

### Flow cytometry

In this study, flow cytometry was used to measure apoptosis. After cells (EDTA-free 0.25% trypsin-digested) from each treatment group were collected, they were washed with PBS three times and resuspended in 100 µL of buffer. At 25 °C, the cells were coincubated with 5 µL Annexin V-FITC and 5 µL PI (BD Biosciences) for 10 min. Finally, after adding the termination buffer, the apoptosis rate was determined by flow cytometry (BD FACSCalibur, USA).

### Reactive oxygen species (ROS) detection

The experimental procedure is described in a previous article (Gu et al. 2019). ROS levels in the cells were determined using an ROS detection kit (Abcam, UK). The cells were mixed with 5 µL of DCFH-DA and incubated at 37 °C for 30 min. The cells were rinsed with fresh medium and imaged under a fluorescence microscope (488 nm excitation).

### Malondialdehyde (MDA) detection

As described previously (Bahr et al. 2019), we used the MDA kit (Beyotime, Shanghai, China) for the detection of MDA in sample cells or mouse retinal tissue. The retinal tissues and cells were lysed and then centrifuged at 1600 × g for 10 min, and the supernatant was taken for subsequent determination. Then, 0.1 mL of lysate was added to the centrifuge tube as a blank control. The samples to be tested and the corresponding reagents were added according to the instructions, mixed well, heated for 15 min using a PCR instrument at 100 °C and cooled at 25 °C in a water bath. Subsequently, the samples were centrifuged for 10 min (1000 g, 25 °C), transferred to 200 µL of supernatant in a black 96-well plate, and placed into a 37 °C microplate reader for detection of absorbance (excitation wavelength 532 nm).

### Fe^2+^ detection

In this study, we used an iron assay kit to detect the content of Fe^2+^ in cells (Fan et al. 2022). The solution and samples were prepared according to the experimental requirements, and the standard and reaction wells were set up (standard wells = 100 µL standard dilution, sample wells were added with 50 µL samples and diluted to 100 µL/well with iron assay buffer). L assay buffer, mixed and incubated (37 °C) for 30 min, 100 µL iron probe was added, mixed and incubated again (37 °C, protected from light) for 60 min and absorbance at 593 nm was measured in a microplate reader.

### Western blot

Western blotting was carried out according to previous studies (Fan et al. 2022). In this study, total protein was extracted from cells and mouse retinal tissue using RIPA lysis buffer containing protease and phosphatase inhibitors, and its concentration was determined by BCA (Sangon Biotech, Shanghai, China). The protein sample was mixed with the loading buffer and denatured at 95 °C for 10 min. Then, the same amount of protein (30 µg/lane) was separated using 10% SDS‒PAGE electrophoresis and transferred to a polyvinylidene fluoride (PVDF) membrane. The membrane was blocked with 5% BSA in TBS in the presence of Tween-20 (0.05%) for 1 h. Subsequently, primary antibodies (the antibodies were purchased from Abcam, and the antibody dilution concentration was 1:1000) were added: Sestrin2, GPX4 (1:2000), FTH1, xCT, HO-1, cleaved-caspase3, BAX, BCL-2, ATF4, CHOP (Thermo Fisher, USA), XBP-1, GRP78, p-GRP78, LC3BII/I (1:2000), Beclin1 (1:2000), and P62, and incubated overnight at 4 °C. The next day, the primary antibody was removed, and the proteins were washed three times with membrane wash buffer for 5 min each. Secondary antibody (1:1000, Abcam, UK) was added and incubated for 2 h at 4 °C, and TBST buffer was used to wash the PVDF membranes. The control protein was β-actin. Subsequently, chemiluminescent reagents were added, and the bands were analyzed for grayscale values using ImageJ software.

### Immunofluorescence staining

ARPE-19 cells were fixed with 4% paraformaldehyde for 30 min, permeated with 0.5% Triton X-100 for 20 min, and then blocked with 5% bovine serum albumin at room temperature for 1 h. Cells were incubated with primary antibodies against GPX4 (1:100) and LC3 (1:200) overnight at 4 °C, washed three times with PBS, and then incubated with fluorescein-labeled secondary antibodies (1:1000) for 2 h. The cells were washed with PBS twice, DAPI (Invitrogen, CA, USA) was added, and the cells were incubated for 15 min in the dark for re-staining. Finally, the slides were sealed with anti-fluorescence quencher. The cells were observed by fluorescence microscopy (Eclipse 80i, Nikon, Japan).

### Laboratory animals

In this study, 70 female C57BL/6 mice aged 7–8 weeks (purchased from the Animal Experimental Center of Kunming Medical University) were selected as experimental animals. Mice were given free access to food and water under SPF conditions, a temperature of 22–26℃, a relative humidity of 52–58%, and a light-dark cycle of 12 h/12 h. After one week of adaptation, the experiment was carried out. The mice were randomly divided into the NC group (the control group ate a normal diet without any treatment; n = 10), DM group (diabetic group, intraperitoneal injection of STZ into mice induced diabetes; n = 15), DM + oe-Sestrin2 group (2 µg oe-Sestrin2 was injected into diabetic mice intravitreally; n = 15), DM + oe-Sestrin2 + erastin group (DM + oe-Sestrin2-treated mice were intraperitoneally injected with 20 mg/kg of the ferroptosis activator erastin three times a week for 4 weeks (Menon et al. 2022); n = 15) and DM + oe-Sestrin2 + 3-MA group (DM + oe-Sestrin2-treated mice were intraperitoneally injected with 15 mg/kg of the autophagy inhibitor 3-methyladenine (3-MA) three times a week for 4 weeks (Bo et al. 2020); n = 15). Diabetes was induced by intraperitoneal injection of 60 mg/kg streptozotocin (STZ) for 5 consecutive days (Suvas et al. 2020; Zhang et al. 2009). Blood glucose was monitored on the 7th day, and the model was established when blood glucose was ≥ 16.7 mmol/L, and used for treatment experiments in the STZ-treated groups. Two months after the successful establishment of the diabetes model, the mice were euthanized by intraperitoneal injection of pentobarbital sodium, and the eyeballs of the mice were collected for subsequent experiments. All animal experimental protocols were approved by the Animal Ethics Committee of Kunming Medical University (approval number: kmmu20211334).

### HE staining

After killing the mice, the eyeballs of the mice were quickly removed and immersed in FAS eyeball fixation solution (Servicebio, Wuhan, China) for 24 h. The sections were then dehydrated with 75%, 85%, 90% and 95% alcohol and anhydrous ethanol. After clearing in xylene, tissues were embedded in paraffin wax and sectioned at 4 μm. Sections were dewaxed with xylene, re-hydrated with graded ethanol, and washed once with distilled water. The samples were stained with hematoxylin for 1 min, rinsed with tap water, differentiated with 1% hydrochloric acid for 10 s, hydrated with 1% ammonia for 5 s, and stained with eosin for 2 min. Finally, the tissues were dehydrated and cleared, sealed with neutral gum, observed and photographed under an optical microscope (BX53, Olympus, Japan).

### TUNEL staining

After xylene dewaxing and gradient alcohol hydration, mouse retinal sections were incubated at 37 °C with 100 µL of protease for 30 min. Then, 50 µL of TdT enzymatic reaction solution was incubated at 37 °C for 1 h away from light. After washing with PBS 3 times, the sections were incubated with 50 µL streptavidin-TRITC solution at 37 °C for 30 min away from light. After washing with PBS three times, the nuclei were re-stained with DAPI staining solution, and the apoptotic cells were observed by fluorescence microscopy after the slides was sealed.

### Statistics and analysis

The data were analyzed and plotted using GraphPad Prism 8. The experimental data are presented as the mean ± standard deviation. Student’s *t*-test was used for comparisons between two groups, one-way analysis of variance (ANOVA) and Tukey’s post hoc tests were used for comparisons among multiple groups with *P* < 0.05 considered as statistically significant.

## Result

### Sestrin 2 inhibits high glucose-induced ferroptosis in ARPE-19 cells

**Fig. 1:**
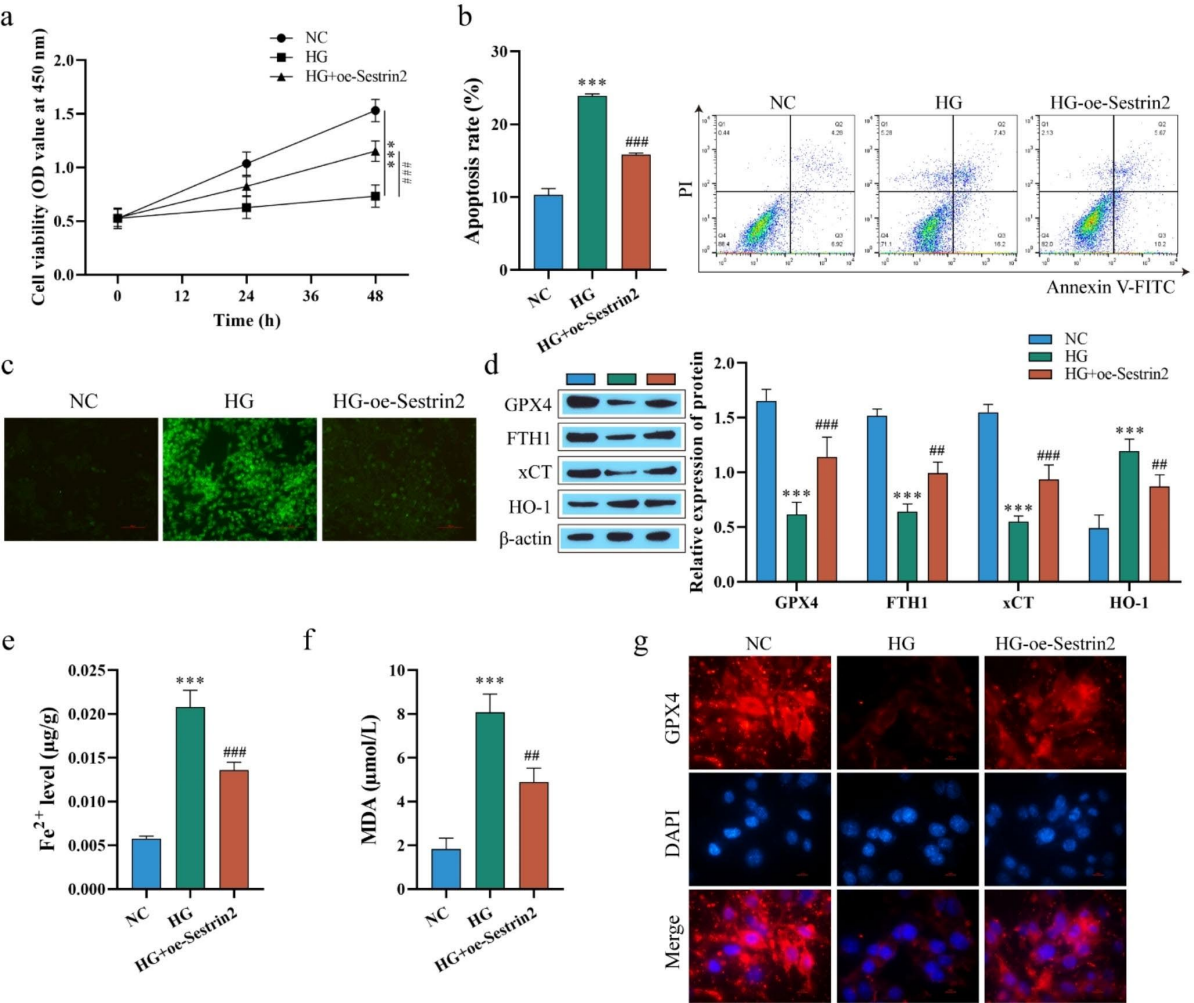
Effect of Sestrin 2 on HG-induced ferroptosis in ARPE-19 cells. **a**: ARPE-19 cell viability was measured by CCK-8; **b**: ARPE-19 apoptosis was detected by flow cytometry; **c**: DCFH-DA assay was used to detect ARPE-19 cells ROS content; Scale bar, 100 μm. **d**: Western blot to detect the expression of ferroptosis-related proteins GPX4, FTH1, xCT and HO-1; **e**: Kit assay to detect Fe^2+^ content; **f**. MDA level was tested; **g**: The expression of GPX4 was detected by immunofluorescence staining. Scale bar, 10 μm. ARPE-19 cells were transfected oe-Sestrin2 and induced with a high glucose concentration (HG, 25 mM) for 48 h. oe-Sestrin2 could increase the viability of ARPE-19 cells and inhibit cell ferroptosis.^* * *^P < 0.001 vs. NC, ^##^*P* < 0.01, ^###^*P* < 0.001 vs. HG

To explore the effect of Sestrin2 on DR, we used HG-induced ARPE-19 cells to construct a cell model of DR and transfected oe-Sestrin2 (overexpression of Sestrin2) into HG-induced ARPE-19 cells. Western blot analysis of the overexpression efficiency of Sestrin2 showed that compared with the NC group, the expression of Sestrin2 was significantly increased after transfection with oe-Sestrin2 (Fig.S1a). Cell viability was determined by CCK-8, and the results showed that HG decreased cell viability. After overexpression of Sestrin2, the cell viability was significantly higher than that in the HG group (Fig. 1a). Flow cytometry was used to detect apoptosis, and the apoptosis rate results were opposite to the cell viability results (Fig. 1b). DCFH-DA assay was used to detect ROS content. The ROS content also showed the same trend as apoptosis (Fig. 1c). In addition, western blot detection of ferroptosis-related proteins showed that GPX4, FTH1 and xCT protein levels were decreased in the HG-treated group, whereas HO-1 expression was increased, and transfection of oe-Sestrin2 attenuated the effect of HG (Fig. 1d). Similarly, Fe^2+^ and MDA levels were significantly increased after HG treatment, while their levels were significantly decreased after transfection with oe-Sestrin2 compared with the HG group (Fig. 1e and f). Finally, the expression of GPX4 was detected by immunofluorescence staining, and the number of GPX4-positive cells decreased in the HG group and increased after transfection with oe-Sestrin2 (Fig. 1g). The above results indicated that overexpression of Sestrin2 could increase the viability of HG-treated ARPE-19 cells, inhibit cell apoptosis, reduce ROS accumulation and Fe^2+^ and MDA levels, and then inhibit cell ferroptosis.

### Sestrin 2 inhibits STZ-induced DR damage in mice

**Fig. 2:**
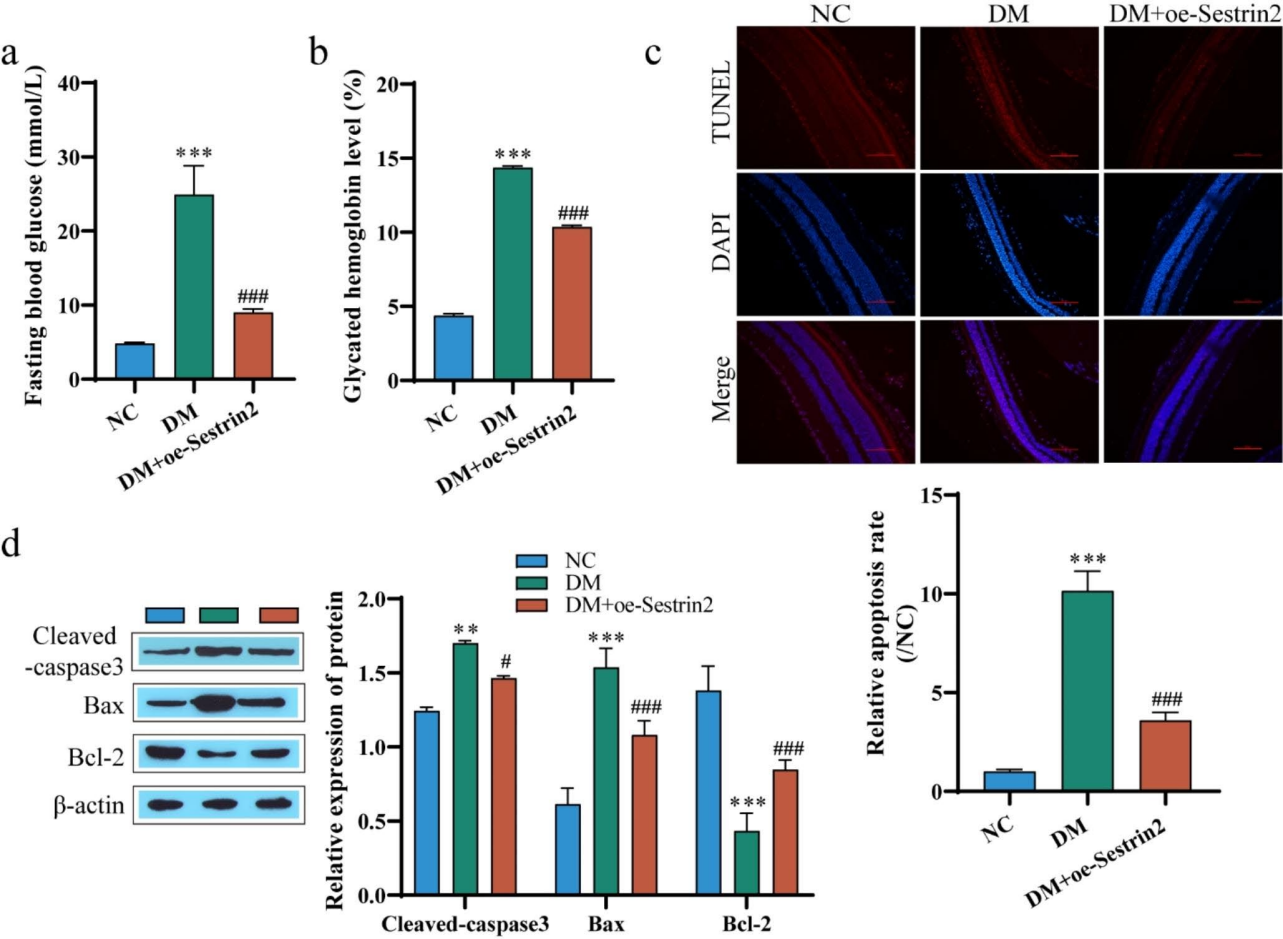
Sestrin2 inhibits STZ-induced DR damage in mice. **a**: Blood glucose determination in mice; b: Detection of glycated hemoglobin level in mice; c: TUNEL staining to detect apoptosis of retinal tissue cells; Scale bar, 100 μm. d: Western blot to detect the expressions of apoptosis-related proteins cleaved-caspase3, BAX and BCL-2. oe-Sestrin2 was injected into diabetic mice and DR damage was detected two months after diabetes induction. oe-Sestrin2 could alleviate retinal damage in diabetic mice. ^* *^P < 0.01,^* * *^P < 0.001 vs. NC; ^#^*P* < 0.05, ^###^*P* < 0.001 vs. DM

To confirm the regulatory effects of Sestrin2 on DR damage in vivo we examined STZ-treated mice as a DR animal model. The blood glucose level of the mice was detected, and compared with the NC group, the blood glucose of DM mice increased significantly and was greater than 16.7 mmol/L. Glycated hemoglobin is an important standard for diabetes diagnosis and treatment monitoring, and the increase of glycated hemoglobin can promote the frequency of DR lesions (Liu et al. 2022c). And the glycated hemoglobin level was also significantly increased in the DM group. However, after overexpression of Sestrin2, the blood glucose and glycated hemoglobin levels of mice were significantly reduced (Fig. 2a and b). HE staining results showed that the retinal tissue structure and cell layer of mice in the normal group were complete and clear, and the cells were arranged orderly, while the retinal thickness of mice in the DM group was thinner, and the cells in the inner and outer nuclear layers were arranged sparsely and disordered. After overexpression of Sestrin2, the degree of retinopathy was reduced, and the thickness has increased to a certain extent (Fig. S2a). To confirm whether Sestrin2 alleviates retinal damage by reducing retinal cell apoptosis, TUNEL staining was performed on mouse retinal tissue, and the results showed that compared with the NC group, the number of apoptotic cells was increased in the DM group, and overexpression of Sestrin2 decreased the number of apoptotic cells (Fig. 2c). Further detection of apoptosis-related proteins in retinal tissues showed that compared with the NC group, the expression of BAX and cleaved-caspase3 proteins in the DM group was significantly upregulated, and the expression of BCL-2 protein was significantly downregulated. Overexpression of Sestrin2, appeared to ameliorate the effect of STZ (Fig. 2d). In conclusion, Sestrin2 can inhibit apoptosis and alleviate retinal damage in diabetic mice.

### Sestrin2 affects ferroptosis by inhibiting STAT3 phosphorylation

**Fig. 3:**
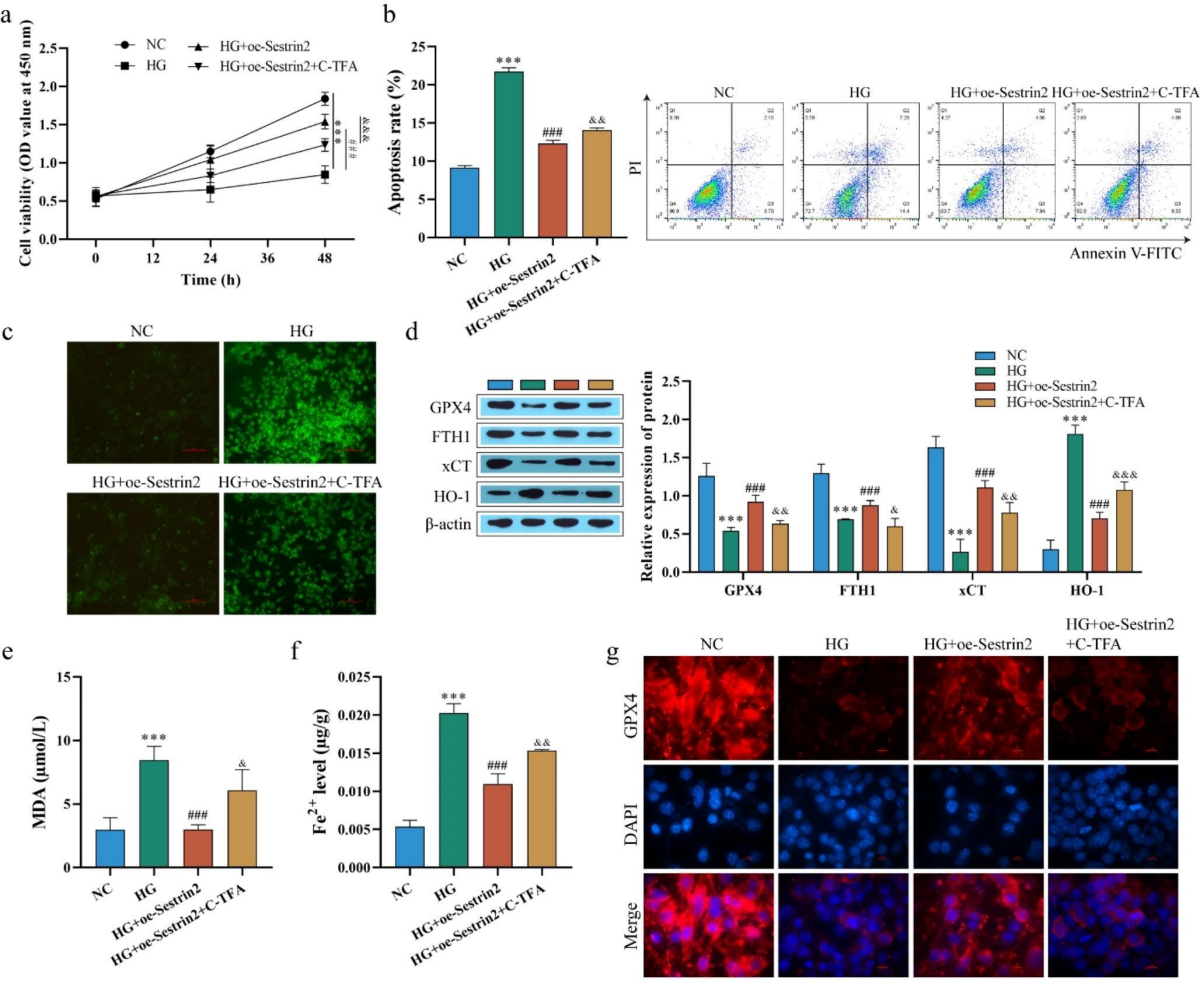
Sestrin2 inhibits STAT3 phosphorylation and ferroptosis. **a**: CCK-8 to detect ARPE-19 cell viability; **b**: flow cytometry to detect ARPE-19 apoptosis; **c**: ROS level was detected by DCFH-DA assay; Scale bar, 100 μm. **d**: The expression of GPX4, FTH1, xCT and HO-1 was detected by western blot; **e**: MDA levels were detected with the kit; **f**: the level of Fe^2+^ was detected; **g**: The expression of GPX4 was detected by immunofluorescence staining. Scale bar, 10 μm. ARPE-19 cells were treated with STAT3 phosphorylation activator C-TFA and Sestrin2 inhibited ferroptosis by inhibiting STAT3 phosphorylation. ^* * *^ P < 0.001 vs. NC; ^###^*P* < 0.001 vs. HG; ^&^P < 0.05, ^&&^ P < 0.01, ^&&&^P < 0.001 vs. HG + oe-Sestrin2

Activation of transcription 3 (STAT3) has also been implicated in oxidative reactions and may be a potential regulator of ferroptosis (Chun et al. 2020). To explore whether Sestrin2 affects ferroptosis through STAT3, we overexpressed Sestrin2 in HG-treated ARPE-19 cells and treated the cells with Colivelin TFA (C-TFA), an activator of STAT3 phosphorylation. CCK-8 detected cell viability, and the results showed that after transfection with oe-Sestrin2, the cell viability was significantly higher than that in the HG group. The effect of oe-Sestrin2 was weakened, and the cell viability was decreased after treatment with C-TFA (Fig. 3a). By contrast, C-TFA increased the apoptosis rate and ROS levels (Fig. 3b-c). In addition, western blot analysis of ferroptosis-related proteins showed that C-TFA reversed the regulatory effect of oe-Sestrin2 on GPX4, FTH1 xCT and HO-1 (Fig. 3d). Fe^2+^ and MDA levels were further examined, and both levels decreased significantly after transfection of oe-Sestrin2 compared with the HG group and increased after C-TFA treatment (Fig. 3e and f). Finally, immunofluorescence staining showed that GPX4-positive cells were markedly increased after transfection of oe-Sestrin2 compared with the HG group, and GPX4-positive cells were decreased after C-TFA treatment (Fig. 3g). These data suggest that Sestrin2 inhibits ferroptosis by inhibiting STAT3 phosphorylation.

### Sestrin2 deficiency activates ferroptosis levels by activating endoplasmic reticulum stress

**Fig. 4:**
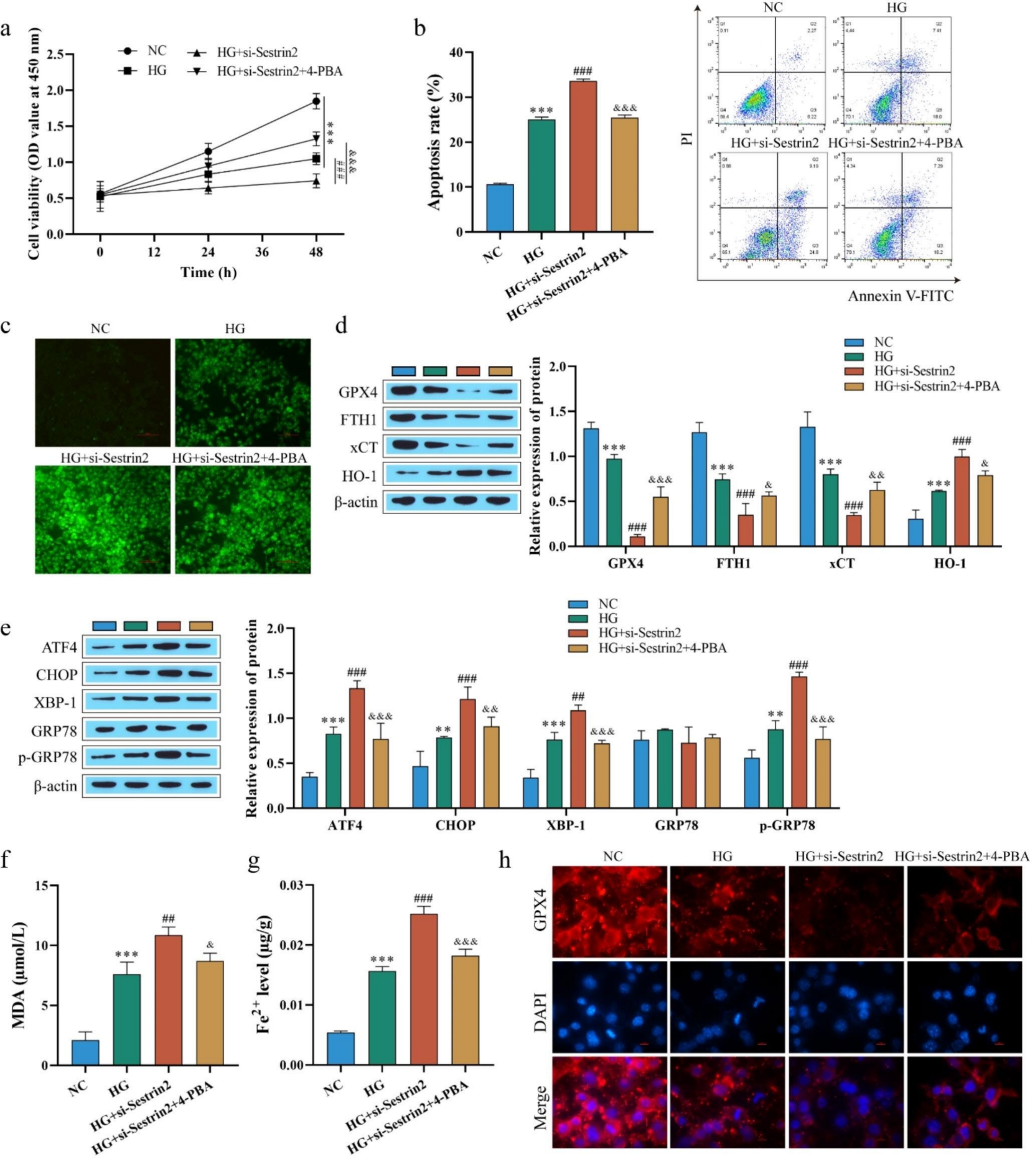
Sestrin2 deficiency activates ferroptosis levels by activating ER stress. **a**-**b**: CCK-8 and flow cytometry were used to detect ARPE-19 cell viability and apoptosis; **c**: ROS level was detected by DCFH-DA assay; Scale bar, 100 μm. **d**-**e**: Ferroptosis-related proteins and ER stress-related proteins levels were detected by western blot; **f**-**g**: Detection of MDA and Fe^2+^ levels with the kits; **h**: The expression of GPX4 was evaluated by immunofluorescence staining. Scale bar, 10 μm. Knockdown Sestrin2 activated ER stress, promoted ferroptosis, ER stress inhibitor 4-PBA reversed the effects of si-Sestrin2. ^* *^P < 0.01, ^* * *^P < 0.001 vs. NC; ^##^*P* < 0.01, ^###^*P* < 0.001 vs. HG; ^&^*P* < 0.05, ^&&^*P* < 0.01 and ^&&&^*p* < 0.001 vs. HG + si-Sestrin2

Studies have demonstrated that ER-associated oxidative stress contributes to the induction of ferroptosis (Wu et al. 2020). In this study, to explore whether Sestrin2 deficiency activates ferroptosis by activating ER stress, Sestrin2 expression was knocked down (si-Sestrin2) in HG-induced cells, and the cells were treated with 4-phenyl butyric acid (4-PBA). 4-PBA is commonly thought to be an “ER stress inhibitor” primarily as a chemical chaperone. The major mechanism for the action of 4-PBA is that the hydrophobic regions of the chaperone interact with exposed hydrophobic segments of the unfolded protein. This interaction protects the protein from aggregation, promotes the folding of proteins, and reduces ER stress (Pao et al. 2021). Western blot analysis of the knockdown efficiency of si-Sestrin2 showed that transfection with si-Sestrin2 significantly decreased the expression of Sestrin2 compared with the NC group (Fig. S1b). CCK-8 detection of cell viability showed that HG treatment reduced cell viability, and after knocking down Sestrin2, cell viability further decreased, but 4-PBA restored cell viability (Fig. 4a). Similarly, si-Sestrin2 further increased HG-induced apoptosis and ROS levels, while 4-PBA reversed the effects of si-Sestrin2 (Fig. 4b-c). Western blot analysis of ferroptosis-related proteins showed that after transfection with si-Sestrin2, the expression of GPX4, FTH1 and xCT proteins was further downregulated, and the expression of HO-1 was further upregulated, while the effect of si-Sestrin2 was weakened after 4-PBA treatment (Fig. 4d). To confirm the occurrence of ER stress, western blotting was used to detect ER stress-related proteins. The results showed that after transfection of si-Sestrin2, the expression of ATF4, CHOP, XBP-1 and p-GRP78 proteins was further upregulated compared with that in the HG group, and the expression of GRP78 protein was not significantly changed. The effect of si-Sestrin2 was weakened after 4-PBA treatment (Fig. 4e). Fe^2+^ and MDA levels were further detected, and both levels were further increased after transfection of si-Sestrin2 compared with the HG group and decreased after 4-PBA treatment (Fig. 4f and g). Finally, immunofluorescence staining showed that the trend of GPX4 expression was consistent with the Western blot results (Fig. 4h). In summary, reduced Sestrin2 levels can activate ER stress, which promotes ferroptosis.

### Sestrin2 inhibits the mTOR signaling pathway and activates autophagy

**Fig. 5:**
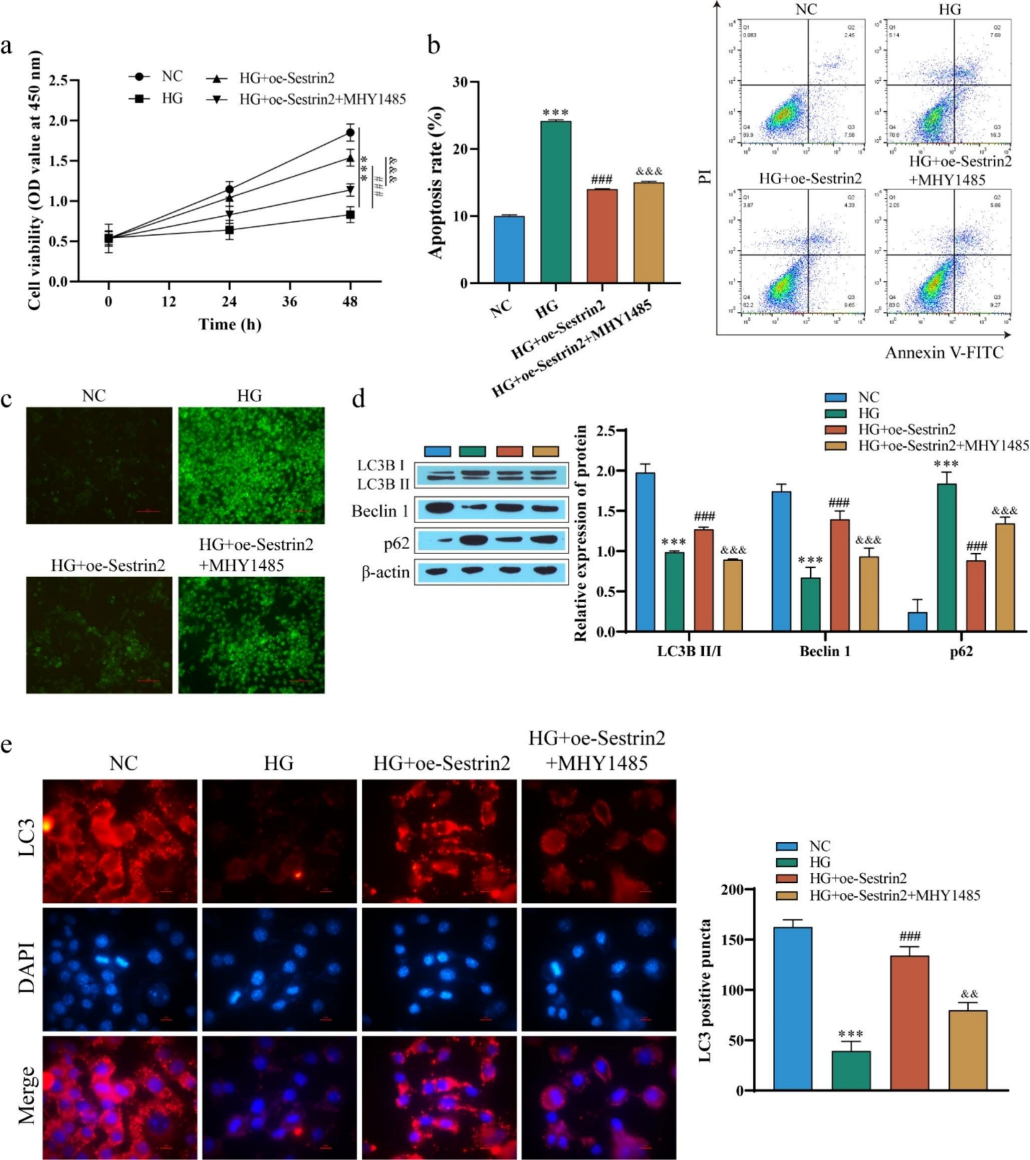
Sestrin2 activates autophagy by inhibiting the mTOR pathway. **a**: ARPE-19 cell viability was determined by CCK-8 assay; **b**: ARPE-19 apoptosis was detected by flow cytometry; **c**: ROS level was detected by DCFH-DA assay; Scale bar, 100 μm. **d**: The expression of autophagy-related proteins LC3, Beclin 1 and p62 was detected by western blotting; **e**: The expression of LC3 was detected by immunofluorescence staining. Scale bar, 10 μm. Sestrin2 inhibited the activation of the mTOR pathway, promoted autophagy and inhibited apoptosis, but the effect of oe-Sestrin2 was reversed after treatment with the mTOR pathway activator MHY1458. ^* * *^P < 0.001 vs. NC; ^###^*P* < 0.001 vs. HG; ^&&^*P* < 0.01, ^&&&^*P* < 0.001 vs. HG + oe-Sestrin2

A potential mechanism underlying the protective effect of Sestrin2 is the induction of autophagy under stress conditions by inhibiting mammalian target of rapamycin (mTOR)(Kim et al. 2015). To verify this possible protective mechanism, we transfected oe-Sestrin2 into HG-induced ARPE-19 cells and treated the cells with the mTOR pathway activator MHY1458. Subsequently, cell viability was measured by CCK-8 assay, and the results showed that after transfection with oe-Sestrin2, the cell viability was significantly higher than that of the HG group, while the cell viability was decreased after treatment with MHY1458 (Fig. 5a). The results of apoptosis were opposite to those of cell viability (Fig. 5b). Measurement of ROS levels in cells revealed that HG treatment increased cellular ROS levels, which were significantly reduced after transfection with oe-Sestrin2, and the effect of oe-Sestrin2 was weakened after MHY1458 treatment (Fig. 5c). Western blot analysis of autophagy-related proteins showed that compared with the NC group, the expression levels of LC3II/I and Beclin1 in cells treated with HG were significantly decreased, while the protein levels of p62 were significantly upregulated. Compared with the HG group, the expression of LC3II/I and Beclin 1 proteins was upregulated, and the expression of p62 was significantly downregulated after transfection with oe-Sestrin2, while the effect of oe-Sestrin2 was reversed after treatment with MHY1458 (Fig. 5d). The results of immunofluorescence staining showed that LC3-positive puncta were significantly increased after oe-Sestrin2 transfection compared with the HG group, while LC3-positive puncta were decreased after MHY1458 treatment (Fig. 5e). These results indicate that Sestrin2 can inhibit the activation of the mTOR pathway, promote autophagy and inhibit apoptosis.

### Sestrin2 inhibits ferroptosis by promoting autophagy

**Fig. 6:**
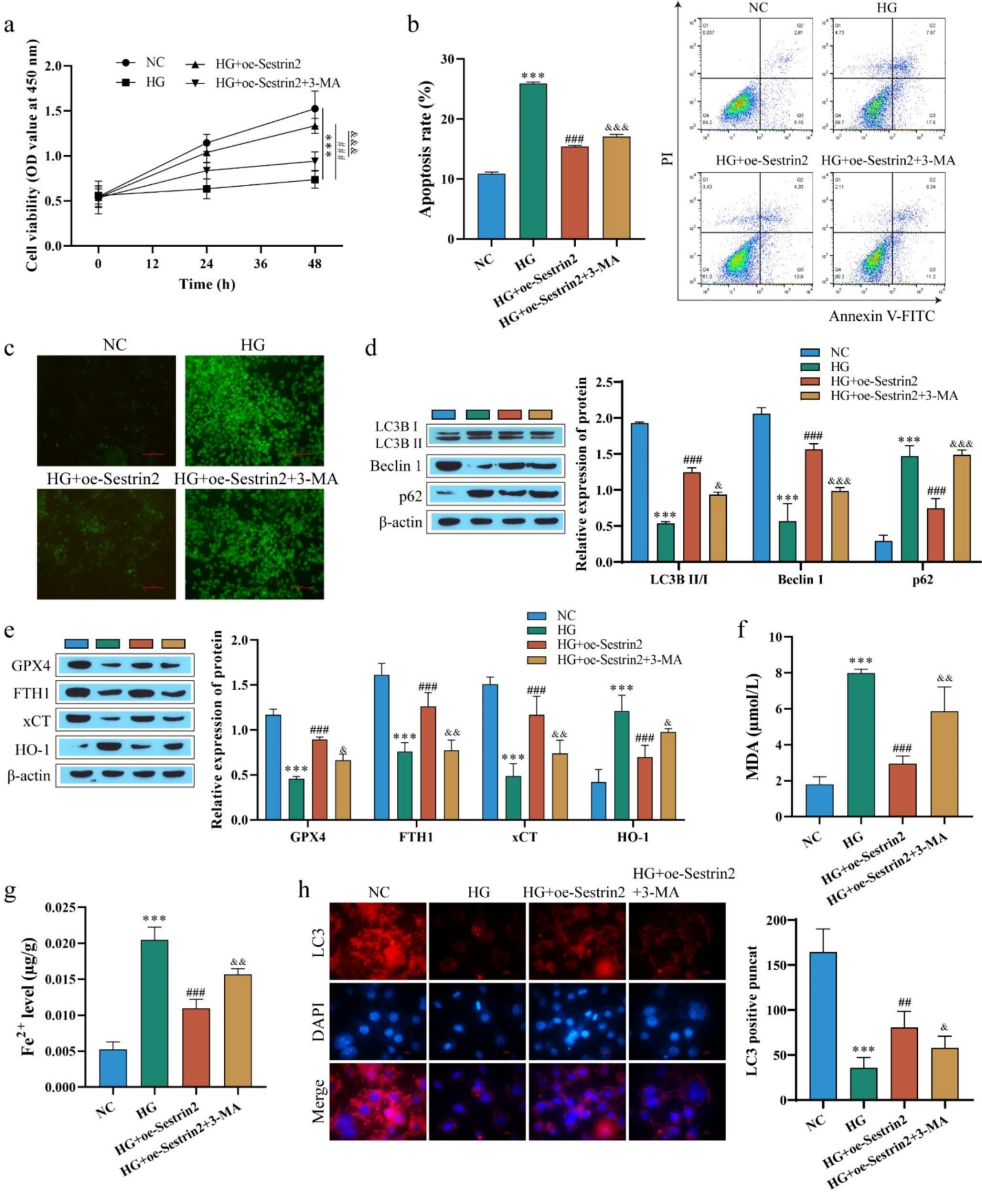
Sestrin2 inhibits ferroptosis by promoting autophagy. **a**: ARPE-19 cell viability was determined by CCK-8 assay; **b**: ARPE-19 apoptosis was detected by flow cytometry; **c**: ROS level was detected by DCFH-DA assay; Scale bar, 100 μm. **d**: The expression levels of LC3, Beclin 1 and p62 were detected by western blot; **e**: The expression levels of GPX4, FTH1, xCT and HO-1 were detected by western blot; **f**: MDA level was detected; **g**: Fe^2+^ level detection; **h**: The expression of LC3 was detected by immunofluorescence staining. Scale bar, 10 μm. Oe-Sestrin2 can promote autophagy and inhibit cell ferroptosis, and the autophagy inhibitor 3-MA treatment reversed the effect of oe-Sestrin2. ^* * *^P < 0.001 vs. NC; ^##^*P* < 0.01, ^###^*P* < 0.001 vs. HG; ^&^*P* < 0.05, ^&&^*P* < 0.01, ^&&&^*P* < 0.001 vs. HG + oe-Sestrin2

It is well known that ferroptosis is regulated by autophagy(Chen et al. 2021b; Lee et al. 2020). In this study, to explore whether Sestrin2 inhibits ferroptosis by promoting autophagy, oe-Sestrin2 was transfected into HG-induced ARPE-19 cells, and the cells were treated with the autophagy inhibitor 3-MA. The levels of autophagy and ferroptosis were then measured. CCK-8 was used to detect cell viability, and the results showed that compared with the HG group, cell viability was significantly increased after transfection with oe-Sestrin2. The cell viability was decreased after 3-MA treatment (Fig. 6a). Next, apoptosis and ROS levels were detected, and the results showed that compared with the HG + oe-Sestrin2 group, the apoptosis rate and ROS levels were increased after 3-MA treatment (Fig. 6b-c). Protein detection results showed that compared with the HG group, oe-Sestrin2 enhanced the expression of LC3II/I and Beclin 1 and decreased the expression of p62, and 3-MA reversed the effect of oe-Sestrin2 (Fig. 6d). Western blot detection of ferroptosis-related proteins showed that after transfection of oe-Sestrin2, GPX4, FTH1 and xCT proteins were significantly upregulated, and HO-1 was significantly downregulated, while 3-MA treatment reversed the effect of oe-Sestrin2 (Fig. 6e). Fe^2+^ and MDA levels were further examined, and levels were significantly decreased after transfection of oe-Sestrin2 compared with the HG group and increased after 3-MA treatment (Fig. 6f and g). For autophagy, LC3-positive puncta were detected again by immunofluorescence staining, and the results showed that after transfection of oe-Sestrin2, LC3-positive puncta increased significantly, and LC3-positive puncta decreased after 3-MA treatment (Fig. 6h). These results suggest that overexpression of Sestrin2 can promote autophagy and inhibit cell ferroptosis.

### Sestrin2 affects ferroptosis and autophagy to mitigate DR progression

**Fig. 7:**
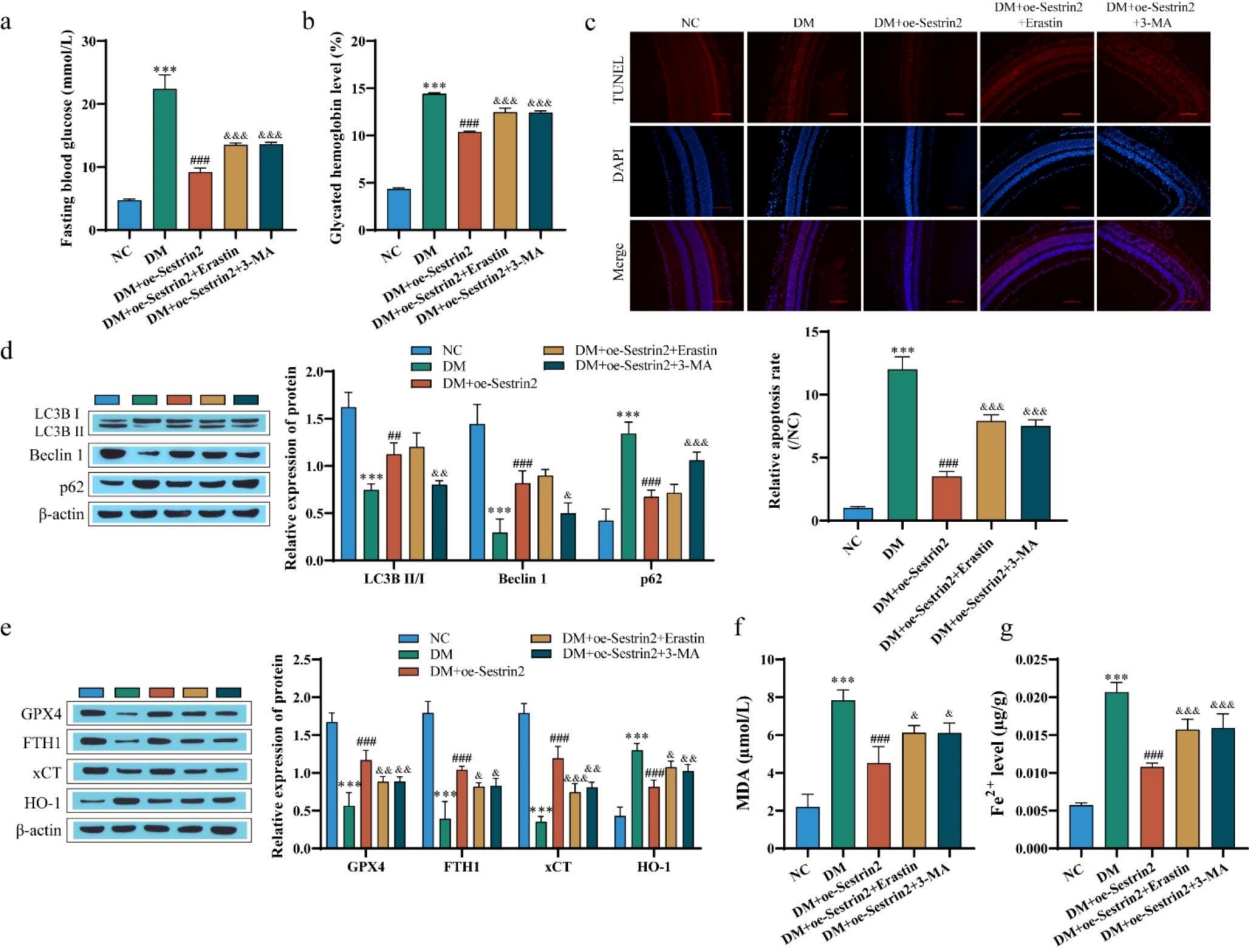
Sestrin2 affects ferroptosis and autophagy to mitigate DR progression. **a**: Blood glucose determination in mice. **b**: Detection of glycated hemoglobin in mice. **c**: Retinal cell apoptosis was detected by TUNEL staining. Scale bar, 100 μm. **d**-**e**: Western blotting was used to detect the expression of autophagy- and ferroptosis-related proteins in mouse retinal tissues. **f**: The MDA level was detected by a kit. **g**: Fe^2+^ levels were detected with the kit. Diabetic mice were intraperitoneally injected with the ferroptosis activator erastin or autophagy inhibitor 3-MA, and oe-Sestrin2 could alleviate DR progression. ^* * *^P < 0.001 vs. NC, ^##^*P* < 0.01, ^###^*P* < 0.001 vs. DM, ^&^*P* < 0.05, ^&&^*P* < 0.01, ^&&&^*P* < 0.001, vs. DM + oe-Sestrin2

DR mice overexpressing Sestrin2 were treated with erastin, an activator of ferroptosis, and 3-MA, an inhibitor of autophagy, to verify in vivo the protective mechanism of Sestrin2 against DR by affecting ferroptosis and autophagy. The blood glucose and glycated hemoglobin levels of mice were detected. The overexpression of Sestrin2 reduced the levels of blood glucose and glycated hemoglobin in STZ-induced mice, and erastin or 3-MA reversed the effect of oe-Sestrin2 (Fig. 7a and b). HE staining showed that the retina of mice in the NC group had good structure and orderly cell arrangement, while the outer layer of the retina of mice in the DM group was deformed and the retinal thickness was significantly reduced compared with that in the NC group. This pathological state was obviously alleviated after overexpression of Sestrin2, while the effect of oe-Sestrin2 was weakened after erastin or 3-MA treatment (Fig. S2b). Similarly, erastin or 3-MA treatment reversed the effects of oe-Sestrin2 on apoptosis, resulting in increased apoptosis rates (Fig. 7c). Western blot analysis of the expression of autophagy-related proteins in tissues showed that compared with the NC group, the expression of p62 protein in the DM group was significantly upregulated, the expression of LC3 II/I and Beclin 1 was significantly downregulated, and the expression of p62 protein was significantly downregulated after overexpression of Sestrin2. The expression of LC3 II/I and Beclin 1 protein was significantly upregulated. 3-MA treatment reversed the effect of oe-Sestrin2, while erastin treatment had no significant effect (Fig. 7d). For the expression of ferroptosis-associated proteins, the expression of GPX4, FTH1, and xCT proteins was upregulated and HO-1 was downregulated after transfection with oe-Sestrin2 compared with the DM group, while the effect of oe-Sestrin2 was weakened after erastin or 3-MA treatment (Fig. 7e). MDA and Fe^2+^ levels were further examined, and both levels were decreased after transfection of oe-Sestrin2 compared with the DM group and increased after erastin or 3-MA treatment (Fig. 7f and g). These data suggest that Sestrin2 alleviates DR progression by promoting autophagy and inhibiting cell ferroptosis.

## Discussion

The pathogenesis of DR is very complex and can cause blindness and affect the daily life of patients in severe cases. As the number of people with diabetes increases, the incidence of DR will gradually increase, which places a heavy burden on the health care system, and current treatments have limited efficacy for patients with DR (Clevers 2006). Therefore, more new targets for the treatment of DR need to be sought. An increasing number of studies have shown that autophagy and ferroptosis are involved in the progression of DR (Tang et al. 2022; Ye et al. 2021). Therefore, this study further investigated how autophagy and ferroptosis play a role in DR, which may provide a potential avenue for the treatment of DR. In this study we used both high glucose-treated ARPE cells and STZ-treated mice to model diabetic conditions and demonstrated that these inhibit autophagy and promote ferroptosis.

Ferroptosis is a recently recognized form of regulatory cell death, and it has been found that inhibiting ferroptosis in age-related macular degeneration is more effective in slowing disease progression than inhibiting apoptosis and necrosis (Sun et al. 2018; Totsuka et al. 2019). Excessive Fe^2+^ in cells exerts a toxic effect and generates a large amount of ROS in vivo through the Fenton reaction, which in turn oxidizes cell membrane lipids and causes ferroptosis (Shen et al. 2018). During ferroptosis, glutathione peroxidase (GPX4) activity is reduced, and GPX4 is the only enzyme in the body that effectively reduces lipid peroxides in biofilms (Chen et al. 2023). In addition, ferritin heavy chain 1 (FTH1), cysteine/glutamate transporter (xCT), and heme oxygenase-1 (HO-1) play important roles in maintaining cellular iron balance during ferroptosis(Ryter 2021; Tian et al. 2020). Consistent with a potential role for ferroptosis in DR, this study found significantly upregulated levels of Fe^2+^ and ROS in HG-induced ARPE-19 cells. HG-treated ARPE-19 cells also showed increased levels of MDA, which is a natural product of lipid oxidation in organisms. Furthermore, the expression of the ferroptosis-related proteins GPX4, FTH1 and xCT was downregulated, but HO-1 was upregulated. These results indicate that HG induced ferroptosis in ARPE-19 cells. Sestrin2 is an important cellular stress protein, and shown to regulate cellular ferroptosis (Park et al. 2019). We showed that overexpression of Sestrin2 alleviates HG-induced ferroptosis damage in ARPE-19 cells, suggesting that Sestrin2 alleviates the progression of DR by inhibiting ferroptosis in ARPE-19 cells.

The endoplasmic reticulum (ER), in addition to its important roles in protein folding and lipid synthesis, is important in regulation of ferroptosis during ER stress (Xie et al. 2016). A variety of ER stress markers are significantly upregulated in DR and are involved in retinal inflammation and microvascular dysfunction in DR (Elmasry et al. 2018; Li et al. 2009; Zhang et al. 2011). Thus, we speculated that Sestrin2 might affect ferroptosis in ARPE-19 cells through ER stress. We demonstrated that, in contrast to over-expression of Sestrin2, knockdown of Sestrin2 promoted the expression of the ER stress-related proteins ATF4, CHOP, XBP-1 and p-GRP78 and at the same time promoted ferroptosis, further aggravating the damage of HG to ARPE-19 cells. However, treatment with the ER stress inhibitor 4-PBA alleviated the cell damage caused by Sestrin2 knockdown. These results suggest that Sestrin2 inhibits ferroptosis in ARPE-19 cells by inhibiting the activation of ER stress. In addition, STAT3 is an important transcription factor in disease progression. It has been found that the activation of STAT3 can inhibit the expression of enzymes required for ferroptosis, and increasing evidence has shown its important role in cell ferroptosis (Brown et al. 2017; Linher-Melville and Singh 2017; Liu and Wang 2019). In addition, phosphorylation of STAT3 was found to be elevated in DR rats (Xu et al. 2018). We postulated that Sestrin2 may trigger cell ferroptosis through STAT3 phosphorylation. We found that treating cells with C-TFA, a phosphorylated activator of STAT3, weakened the protective effect of Sestrin2 on cells, as shown by increased the levels of Fe^2+^, MDA and ROS in cells, and promoted cell ferroptosis. This suggests that Sestrin2 inhibits cell ferroptosis by inhibiting STAT3 phosphorylation.

Autophagy plays a dual role in DR, playing a role in its occurrence and deterioration. Autophagy activity can promote cell survival under mild stress, while dysregulation of autophagy can lead to cell death under severe stress (Dehdashtian et al. 2018). Studies have found that autophagy can regulate ROS levels, and excessive accumulation of ROS leads to oxidative stress and mitochondrial dysfunction, thus promoting the initiation of autophagy (Li et al. 2015). To identify changes in autophagy levels in DR, we assessed changes in expression of autophagy-associated proteins and found that the expression of LC3II/I and Beclin1 in HG-induced ARPE-19 cells was significantly downregulated, whereas the expression of p62 was significantly upregulated, indicating that high glucose inhibited autophagy. As mTOR affects the progression of DR (Casciano et al. 2022) and Sestrin2 has been implicated in induction of autophagy under stress conditions by inhibiting mTOR (Kim et al. 2015; Kim and Guan 2015), we examined the effects of activating the mTOR pathway on cells over-expressing Sestrin2. Treating ARPE-19 cells with MHY1458, which activates mTOR pathway reversed the protective effects of Sestrin2 over-expression. This suggests that Sestrin2 is involved in the positive regulation of autophagy by inhibiting mTOR pathway activation.

Studies have found that autophagy regulates the process of ferroptosis (Chen et al. 2021b). Autophagy has been identified as an upstream mechanism that induces ferroptosis by regulating cellular iron homeostasis and cell ROS production (Zhang et al. 2020). In our study, treatment with the autophagy inhibitor 3-MA weakened the protective effect of overexpression of Sestrin2 on ARPE-19 cells and promoted cell ferroptosis, indicating that Sestrin2 inhibits cell ferroptosis by activating autophagy. In addition, animal experiment also verified the results of the cell experiment, and it was found that retinal damage occurred in mice in the DR group, and after treatment with Sestrin2, the pathological status of the mouse retina was significantly improved. Notably, when treated with the ferroptosis activator erastin, there was no significant effect on the expression of autophagy-related proteins in mouse retinal tissues, suggesting that Sestrin2 inhibits ferroptosis levels by promoting autophagy, thereby easing the progression of DR.

In summary, high glucose was shown to inhibit ARPE-19 cell autophagy and promote cell ferroptosis. In addition, overexpression of Sestrin2 can enhance the antioxidant capacity of cells, promote autophagy and inhibit cell ferroptosis. The results of this study will provide a new theoretical basis for the treatment of DR.

### Electronic supplementary material

Below is the link to the electronic supplementary material.

Supplementary Material 1

Supplementary Material 2 Supplementary Material 2

Supplementary Material 3

## Data Availability

The datasets used and/or analyzed during the current study are available from the corresponding author upon reasonable request.
