# Correction: Sestrin2 ameliorates diabetic retinopathy by regulating autophagy and ferroptosis

**DOI:** 10.1007/s10735-024-10181-w

**Published:** 2024-01-27

**Authors:** Xiaoting Xi, Qianbo Chen, Jia Ma, Xuewei Wang, Junyan Zhang, Yan Li

**Affiliations:** 1 Ophthalmology Department, The First Affiliated Hospital of Kunming Medical University, Kunming, 650032 Yunnan China; 2 Department of Clinical Epidemiology and Evidence-Based Medicine, Shanxi Bethune Hospital, Shanxi Academy of Medical Sciences, Taiyuan, 030000 Shanxi China

**Correction to: Journal of Molecular Histology** 10.1007/s10735-023-10180-3

In the original article, supplementary figure was missing and has now been uploaded.

