# Supplementary material for: Sestrin2 ameliorates diabetic retinopathy by regulating autophagy and ferroptosis
Source: J Mol Histol. 2024 Jan 2;55(2):169–84. doi: 10.1007/s10735-023-10180-3 (PMC10991044; doi:10.1007/s10735-023-10180-3)
Supplement: Supplementary file 1 — Supplementary Material 1 [file 10735_2023_10180_MOESM1_ESM.docx]

OE-Sestrin2

5‘-ATGATCGTGGCGGACTCCGAGTGCCGCGCAGAGCTCAAGGACTACCTGCGGTTCGCCCCGGGCGGCGTCGGCGACTCGGGCCCCGGAGAGGAGCAGAGGGAGAGCCGGGCTCGGCGAGGCCCTCGAGGGCCCAGCGCCTTCATCCCCGTGGAGGAGGTCCTTCGGGAGGGGGCTGAGAGCCTCGAGCAGCACCTGGGGCTGGAGGCACTGATGTCCTCTGGGCGAGTAGACAACCTGGCAGTGGTGATGGGCCTGCACCCTGACTACTTTACCAGCTTCTGGCGCCTGCACTACCTGCTGCTGCACACGGATGGTCCCTTGGCCAGCTCCTGGCGCCACTACATTGCCATCATGGCTGCCGCCCGCCATCAGTGTTCTTACCTGGTAGGCTCCCACATGGCCGAGTTTCTGCAGACTGGTGGTGACCCTGAGTGGCTGCTGGGCCTCCACCGGGCCCCCGAGAAGCTGCGCAAACTCAGCGAGATCAACAAGTTGCTGGCGCATCGGCCATGGCTCATCACCAAGGAACACATCCAGGCCTTGCTGAAGACCGGCGAGCACACTTGGTCCCTGGCCGAGCTCATTCAGGCTCTGGTCCTGCTCACCCACTGCCACTCGCTCTCCTCCTTCGTGTTTGGCTGTGGCATCCTCCCTGAGGGGGATGCAGATGGCAGCCCTGCCCCCCAGGCACCTACACCCCCTAGTGAACAGAGCAGCCCCCCAAGCAGGGACCCGTTGAACAACTCTGGGGGCTTTGAGTCTGCCCGCGACGTGGAGGCGCTGATGGAGCGCATGCAGCAGCTGCAGGAGAGCCTGCTGCGGGATGAGGGGACGTCCCAGGAGGAGATGGAGAGCCGCTTTGAGCTGGAGAAGTCAGAGAGCCTGCTGGTGACCCCCTCAGCTGACATCCTGGAGCCCTCTCCACACCCAGACATGCTGTGCTTTGTGGAAGACCCTACTTTCGGATATGAGGACTTCACTCGGAGAGGGGCTCAGGCACCCCCTACCTTCCGGGCCCAGGATTATACCTGGGAAGACCATGGCTACTCGCTGATCCAGCGGCTTTACCCTGAGGGTGGGCAGCTGCTGGATGAGAAGTTCCAGGCAGCCTATAGCCTCACCTACAATACCATCGCCATGCACAGTGGTGTGGACACCTCCGTGCTCCGCAGGGCCATCTGGAACTATATCCACTGCGTCTTTGGCATCAGATATGATGACTATGATTATGGGGAGGTGAACCAGCTCCTGGAGCGGAACCTCAAGGTCTATATCAAGACAGTGGCCTGCTACCCAGAGAAGACCACCCGAAGAATGTACAACCTCTTCTGGAGGCACTTCCGCCACTCAGAGAAGGTCCACGTGAACTTGCTGCTCCTGGAGGCGCGCATGCAAGCCGCTCTGCTGTACGCCCTCCGTGCCATCACCCGCTACATGACCTGA-3’

si-Sestrin2

SS Sequence：GCGCAGAGCUCAAGGACUACC

AS Sequence：UAGUCCUUGAGCUCUGCGCGG
