# Supplementary figures and images for: Sestrin2 ameliorates diabetic retinopathy by regulating autophagy and ferroptosis
Source: J Mol Histol. 2024 Jan 2;55(2):169–84. doi: 10.1007/s10735-023-10180-3 (PMC10991044; doi:10.1007/s10735-023-10180-3)

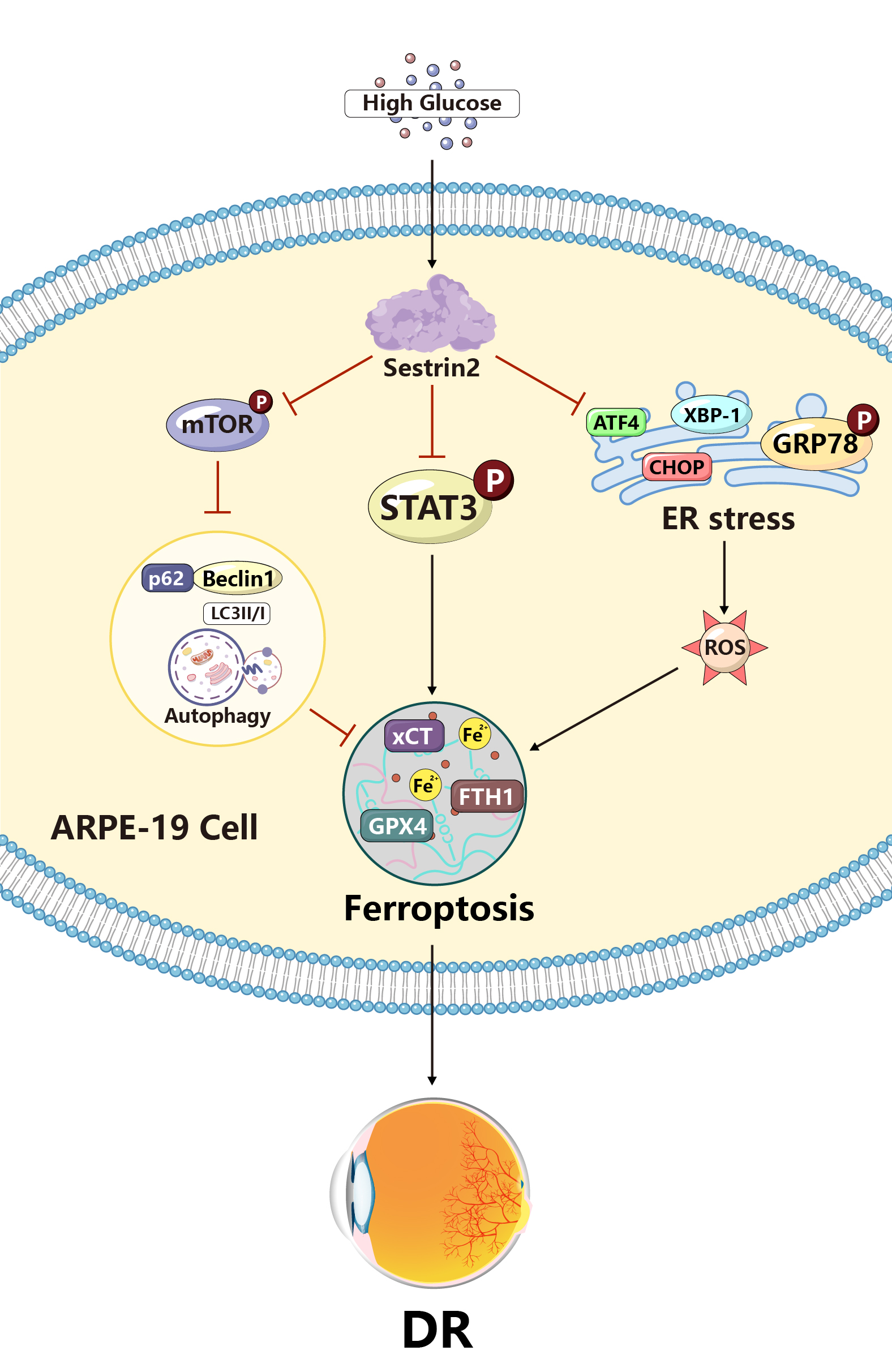

Supplement: Supplementary file 2 — Supplementary Material 2 [file 10735_2023_10180_MOESM2_ESM.jpg]
