# Supplementary material for: Sestrin2 ameliorates diabetic retinopathy by regulating autophagy and ferroptosis
Source: J Mol Histol. 2024 Jan 2;55(2):169–84. doi: 10.1007/s10735-023-10180-3 (PMC10991044; doi:10.1007/s10735-023-10180-3)

**Figure legends**

Figure S1 Transfection efficiency detection of Sestrin2. **a**: The overexpression efficiency of Sestrin2 was detected by western blot. **b**: Knockdown efficiency of Sestrin2 was measured by western blot. ^* *^ P < 0.01, ^* * *^ P < 0.001 vs. NC
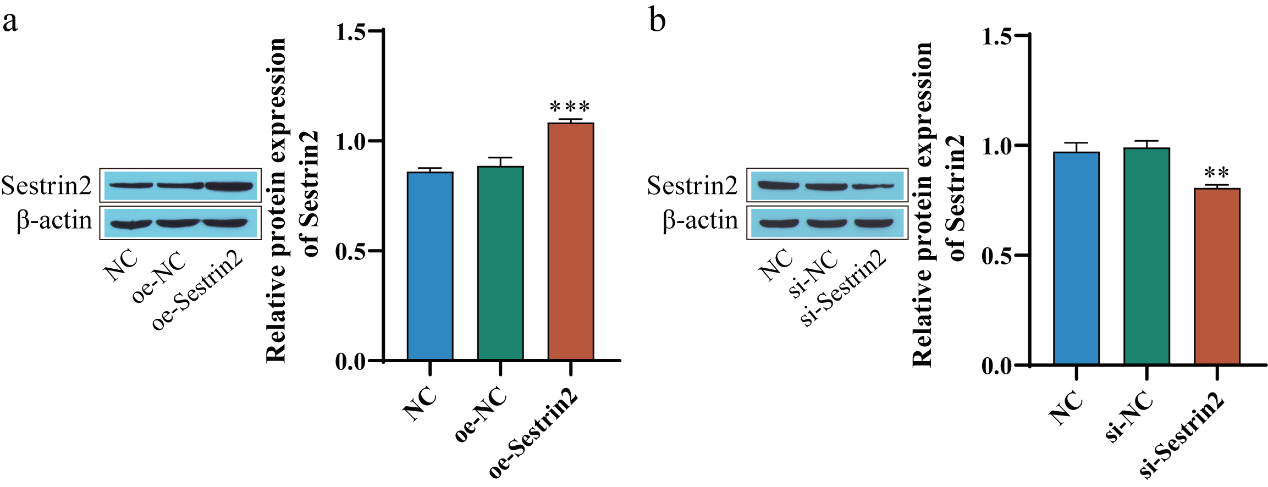


Figure S2 Pathological changes in the mouse retina. **a**-**b**: Pathological changes in the mouse retina were observed by HE staining. Scale bar, 100 μm. ONL: outer nuclear layer; INL: inner nuclear layer; GCL: Ganglion cell layer. ^* * *^ P < 0.001 vs. NC, ^##^ P < 0.01, ^###^ P < 0.001 vs. DM, ^&^ P < 0.05 vs. DM + oe-Sestrin2
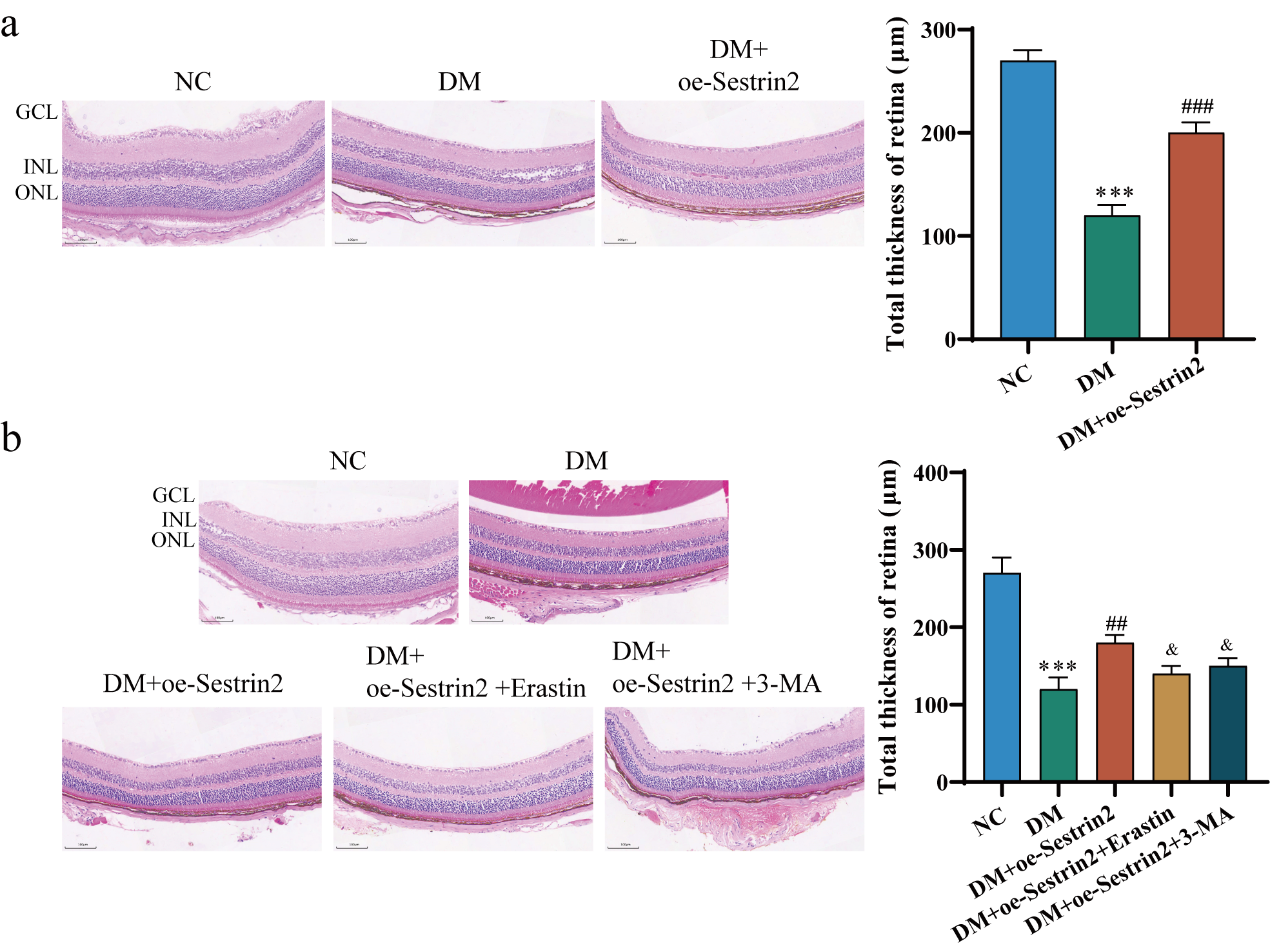

Supplement: Supplementary file 3 — Supplementary Material 3 [file 10735_2023_10180_MOESM3_ESM.docx]
